# Designing an Indicator‑Driven, Value‑Based Architecture for Pneumonia Prevention in Japan: A Formative Policy Viewpoint on Adult Vaccination and Oral Care

**DOI:** 10.2196/86912

**Published:** 2026-02-27

**Authors:** Kazumi Kubota, Ataru Igarashi, Satoko Fujihara, Kenichi Imai

**Affiliations:** 1 Research Organization Shimonoseki City University Yamaguchi Japan; 2 Department of Healthcare Information Management University of Tokyo Hospital Tokyo Japan; 3 Department of Health Policy and Public Health Graduate School of Pharmaceutical Sciences The University of Tokyo Tokyo Japan; 4 Institute for Health Economics and Policy Tokyo Japan; 5 Department of Microbiology and Immunology Nihon University School of Dentistry Tokyo Japan

**Keywords:** adult vaccination, pneumococcal, influenza, oral care, hospital‑acquired pneumonia, cost‑effectiveness, value‑based payment, Japan

## Abstract

Japan's aging society concentrates pneumonia burden across communities, long‑term care, perioperative pathways, and hospitals. Adult pneumococcal and influenza vaccination are supported by trials and meta‑analyses and are often cost‑effective; yet, realized value depends on targeting, measurement, financing, and pairing with bedside prevention such as oral care and hospital‑acquired pneumonia bundles. This viewpoint proposes a formative, indicator‑driven architecture linking cost‑effectiveness to operations by aligning vaccination with complementary oral‑care prevention and value‑based payment under the existing policy infrastructure. Working within the Ministry of Health, Labour and Welfare/Central Social Insurance Medical Council health technology assessment framework, Center for Outcomes Research and Economic Evaluation for Health methods, and claims–electronic health record linkage via My Number insurance card, we specify a compact national indicator set: vaccination coverage and timeliness, nonventilator hospital‑acquired pneumonia, ventilator‑associated pneumonia, postoperative and stroke‑associated pneumonia, antibiotic days of therapy, and length of stay, with pragmatic risk adjustment and present‑on‑admission flags. Value levers first reward reliable reporting and adherence to evidence‑based bundles and then share verified, risk‑adjusted savings. Long‑term care facilities receive add‑ons for professional oral care in high‑risk residents; hospitals receive quality add‑ons and shared savings; perioperative pathways may incorporate oral health management; and stroke units standardize oral hygiene with dysphagia screening. A phased roadmap details the pilot co‑design, governance, risk‑adjusted reporting with equity safeguards, and iterative recalibration by using real‑world evidence. The learning loop—measure, report, improve, generate evidence, adapt cost‑effectiveness, recalibrate payment—converts modeled value into lived experience: fewer pneumonias, reduced antibiotic exposure, shorter stays, improved function, and dignity at favorable or potentially lower costs, context-depending on baseline pneumonia rates, implementation fidelity, and local unit costs.

## Introduction

Japan’s rapidly aging society concentrates the burden of pneumonia across community settings, long‑term care, perioperative pathways, and hospitals. Pneumonia remains among the leading causes of death and hospitalization, with aspiration‑related events common in nursing homes and poststroke care and with hospital‑acquired pneumonia worsening outcomes in wards and in intensive care units (ICUs) [1-4]. Adult vaccination against pneumococcal disease and influenza (flu) reduces disease and complications according to randomized trials and meta‑analyses and is frequently cost‑effective across risk groups [5-8]. Alongside this evidence, Japan has assembled policy infrastructure capable of translating value into practice through a national health technology assessment (HTA) program under Japan's Ministry of Health, Labour and Welfare (MHLW) and the Central Social Insurance Medical Council (Chuikyo), guided by the Center for Outcomes Research and Economic Evaluation for Health (C2H) methods [9,10]. The rollout of the My Number health insurance card and the progress in health information standardization enable claims–electronic health record (EHR) linkages for routine measurement and learning [11].

Economic models persuade at the time of decision; however, value is diluted when uptake is uneven, outcomes are inconsistently measured, or payment recalibration occurs in opaque ways. Realized value from adult vaccination depends on reaching high‑risk cohorts, ensuring timely influenza vaccination, and embedding vaccination within high‑quality bedside prevention. Professional and mechanical oral care lowers pneumonia in nursing homes [12]; perioperative oral health management is associated with reduced postoperative pneumonia and mortality in cancer surgery [13]; and prevention bundles for nonventilator hospital‑acquired pneumonia (NV‑HAP) and ventilator‑associated pneumonia (VAP) improve outcomes when implemented with fidelity [14-16]. Vaccines complement rather than substitute for these elements. In practical terms, the proposed architecture aims to close the implementation gap by making prevention performance visible and comparable (through computable and auditable indicators with present-on-admission [POA] safeguards and pragmatic risk adjustment) and by linking these measures to staged incentives that fit Japan’s existing fee schedule and HTA review cycles. For example, in long-term care, an add-on for professional oral care can be tied to verified delivery in high-risk residents and tracked alongside risk-adjusted aspiration pneumonia and vaccination coverage indicators. In acute and perioperative care, facilities can first be rewarded for reliable reporting and documented adherence to prevention bundles, and only subsequently participate in shared savings once improvements in risk-adjusted outcomes and length of stay are independently validated. This staged “measure → report → improve → verify → recalibrate” loop is intended to translate modeled value into consistent bedside practice while maintaining transparency and equity safeguards. This viewpoint proposes an indicator‑driven, value‑based architecture that connects cost‑effectiveness to operations for pneumonia prevention in Japan. The design is formative and non‑eHealth by intent: it focuses on policy rules, workforce and supplies, computable indicators, and payment incentives that can be implemented with existing digital rails and institutional processes. We draw on Japanese and international evidence, HTA methods, and stakeholder insights, including a recent policy recommendation issued through the Health and Global Policy Institute Expert Policy Advocacy Platform [17]. The goal is to make the link between modeled value and lived experience credible, visible, and improvable.

## Policy Context and Rationale for a Formative Approach

Under Japan’s HTA program, cost‑effectiveness informs pricing and reimbursement while recognizing uncertainty and the need for periodic review [9,10]. Interoperability efforts using the My Number insurance card create the technical and governance conditions to link administrative and clinical data at scale with appropriate safeguards [11,18]. These conditions favor a formative approach that begins with low‑burden, transparent measurement; publishes versioned specifications; and evaluates new payment levers in pilots before national rollout. The approach emphasizes deliverables that de‑risk implementation: indicator logic and change logs, minimum datasets and data flows, a data‑protection impact assessment and standardized data‑use agreements, POA education and audits, and validation protocols that compare algorithm‑flagged cases with clinical reviews in a sample.

### A Value‑Based Logic Model

**Figure 1:**
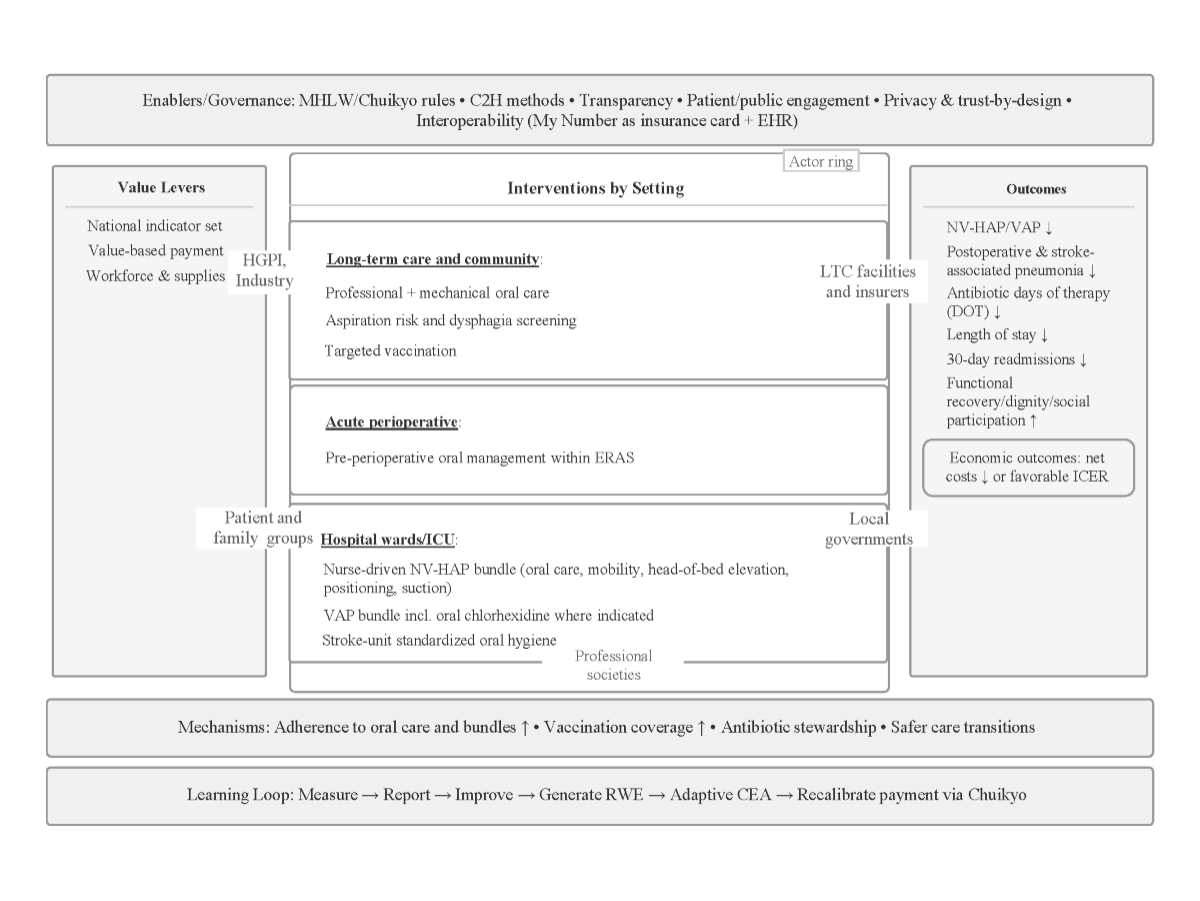
Logic model for value-based pneumonia prevention in Japan. This logic model shows how governance and digital enablers (MHLW/Chuikyo rules, C2H methods, transparency, patient/public engagement, privacy-by-design, and My Number–EHR interoperability) connect value levers (a national indicator set, value-based payment, and workforce/supplies) to setting-specific interventions across long-term care, perioperative care, and hospital wards/ICU. Through improved adherence to oral care and bundles, higher vaccination coverage, antibiotic stewardship, and safer care transitions, the learning loop of measure–report–improve–generate RWE–adaptive CEA–payment recalibration yields measurable gains, including lower NV-HAP/VAP, postoperative and stroke-associated pneumonia, reduced DOT, shorter length of stay, and fewer 30-day readmissions, alongside better function and dignity and favorable costs/ICERs. C2H: Center for Outcomes Research and Economic Evaluation for Health; CEA: cost-effectiveness analysis; Chuikyo: Central Social Insurance Medical Council; DOT: days of therapy; EHR: electronic health record; ICER: incremental cost-effectiveness ratio; ICU: intensive care unit; MHLW: Ministry of Health, Labour and Welfare; NV-HAP: nonventilator hospital-acquired pneumonia; RWE: real-world evidence; VAP: ventilator-associated pneumonia.

Figure 1 summarizes the logic model. Governance and digital enablers include MHLW/Chuikyo rules, C2H methodological guidance, transparency and public engagement, privacy‑ and trust‑by‑design, and interoperability between claims and EHRs [9-11,17,18]. Value levers comprise a national indicator set, value‑based payment, and workforce and supplies. Interventions are setting‑specific. In long‑term care, professional and mechanical oral care complements caregiver‑delivered daily care. In perioperative care, preoperative and perioperative oral health management is incorporated into enhanced recovery pathways and coordinated with surgeons, anesthesiologists, nurses, and dentists [13]. In general wards, nurse‑driven NV‑HAP bundles include oral care, mobility, head‑of‑bed elevation, positioning, and suction as clinically indicated, guided by national toolkits [15,19]. In ICUs, VAP bundles integrate ventilator care, sedation minimization, early mobility, and oral care, with context‑dependent use of oral chlorhexidine aligned to guidelines [14,16]. In stroke units, standardized oral hygiene is coordinated with dysphagia screening and management [20]. Mechanisms operate through higher vaccination coverage and timeliness, better adherence to oral care and bundles, antibiotic stewardship, and safer transitions, leading to reductions in NV‑HAP and VAP, postoperative and stroke‑associated pneumonia, antibiotic days of therapy (DOT), length of stay, and readmissions.

### Ethical Considerations

This viewpoint uses only publicly available sources and proposes a formative policy design without human participant data.

### Indicator Set and Computable Definitions

Because EHR capabilities vary across facilities, specifications are designed to be computable using claims as a baseline, with clearly flagged EHR-based enhancements where available (eg, medication administration records for DOT). Versioned specifications, minimum datasets, and validation (eg, sampled chart review) are used to support comparability while making data limitations explicit. Indicators tie model assumptions to reality and must be computable, comparable, and auditable. We propose a compact suite that can be extracted from routine data with versioned specifications and public change logs. Coverage indicators assess pneumococcal and seasonal influenza vaccination among older adults and prespecified high‑risk cohorts, with timeliness for influenza measured as vaccination before a seasonal cutoff [5-8]. Outcome indicators include pneumonia hospitalizations per 1000 older adults, NV‑HAP per 1000 patient‑days with clear surveillance definitions that exclude POA cases, ventilator‑associated events and pneumonia per 1000 ventilator‑days, 30‑day postoperative pneumonia for specified procedures, and stroke‑associated pneumonia within 7 days of admission or stroke onset [4,13-16,19-21]. Antibiotic consumption is captured as DOT per 1000 patient‑days using the National Healthcare Safety Network antimicrobial use specification, with transparent claims‑based proxies when electronic medication administration records are unavailable [22]. In long‑term care, aspiration pneumonia per 100 resident‑years and, where feasible, antibiotic DOT per 100 resident‑days are adjusted for care‑need level and comorbidities [3,12]. Multimedia Appendix 1 and Multimedia Appendix 2 provide the minimum indicator specifications and the illustrative budget impact scenario (including assumptions and an unfavorable-case scenario) in full.

Operationally, we envision a staged data-flow model. In the pilot phase, indicator computation is performed primarily by facilities or municipalities using standardized, version-controlled specifications, with My Number–enabled linkage used to reconcile claims-based denominators and utilization with EHR time-stamps (eg, antibiotic starts, imaging) under agreed governance; aggregated, deidentified outputs are then submitted for benchmarking. As interoperability and governance mature, the same linkage can support more centralized, automated indicator calculation and auditing.

Fair comparisons require pragmatic risk adjustment. Minimum adjustment includes age, sex, comorbidities, emergency vs elective admission, diagnosis and procedure classes, ward or ICU type, and severity proxies, drawing on established guidance [23]. POA flags protect facilities from penalties for community‑onset disease and interfacility transfers. For stroke‑related outcomes, models incorporate stroke severity and dysphagia screening when recorded [20]. To address misclassification concerns, NV‑HAP algorithms combine coded diagnoses with treatment and imaging signals and are validated against structured chart review in a sample per facility. Public reporting should include uncertainty intervals and peer grouping by case mix to reduce spurious league tables. The minimum specifications are summarized in Multimedia Appendix 1.

### Payment Levers Aligned With Evidence

Payment design can determine whether evidence-based prevention is implemented with fidelity, particularly when reporting burden and front-line workload are high. We therefore adopt a conservative sequence that pays first for verified reporting and adherence and then shares a fraction of risk‑adjusted, independently verified savings. In long‑term care, facilities receive an add‑on for professional and mechanical oral care in high‑risk residents. Evidence from randomized and quasi‑experimental studies indicates that oral care reduces pneumonia incidence and mortality risk in frail older populations [12], and cost structures in Japan make this strategy plausible from a payer perspective. Facilities that improve vaccination coverage and reduce risk‑adjusted aspiration pneumonia rates receive public recognition and, after validation, modest shared savings.

In acute care, hospitals receive add‑ons tied to adherence with NV‑HAP and VAP prevention bundles supported by infection prevention and critical care guidelines [14-16]. Adherence focuses on documented oral care frequency and technique, head‑of‑bed elevation, early mobility, ventilator care, sedation minimization, and suction when indicated. A conservative shared‑savings mechanism returns a fraction of verified risk‑adjusted reductions in pneumonia‑related costs and length of stay relative to a rolling baseline. For perioperative pathways, enhanced‑recovery payments require preoperative and perioperative oral health management for selected procedures, with postoperative pneumonia as a quality gate informed by national database studies linking oral health management with lower postoperative pneumonia and improved survival [13]. For vaccination, modest pay‑for‑performance elements reward providers and municipalities that reach age‑ and risk‑adjusted coverage targets, with additional weight for people with multimorbidity, frailty, or a recent pneumonia. Parameter values are initialized using published effect sizes and Japanese prices and are recalibrated using observed outcomes and net costs after pilots [9,24].

A simple budget impact scenario illustrates fiscal feasibility. This scenario is intended as an illustrative, order-of-magnitude calculation (not a hospital-specific economic evaluation); all parameters and calculation steps are shown in Multimedia Appendix 2. We assume baseline NV-HAP of 1.2 per 1000 patient-days and VAP of 5 per 1000 ventilator-days, with illustrative conservative relative reductions (25% for NV-HAP and 30% for VAP) and excess lengths of stay of 4 and 6 days, respectively. Published implementation studies of oral care–centered NV-HAP prevention bundles have reported larger reductions in NV-HAP incidence than those assumed here, supporting the plausibility of using conservative illustrative effect sizes for an order-of-magnitude example [25]. Under these assumptions, gross savings are approximately ¥8.16 million (US $53,310) with incremental program costs of ¥4.39 (US $28,670) million in year 1; sharing 30% of verified savings still leaves payer-side net savings. Multimedia Appendix 2 also includes an unfavorable-case scenario (lower baseline incidence, smaller effect size, and lower unit costs) showing that the budget impact can approach break-even. This scenario deliberately excludes additional gains from perioperative oral health management and dysphagia screening, which would further improve the budget profile [13,20].

### Phased Implementation Roadmap and Formative Deliverables

**Figure 2:**
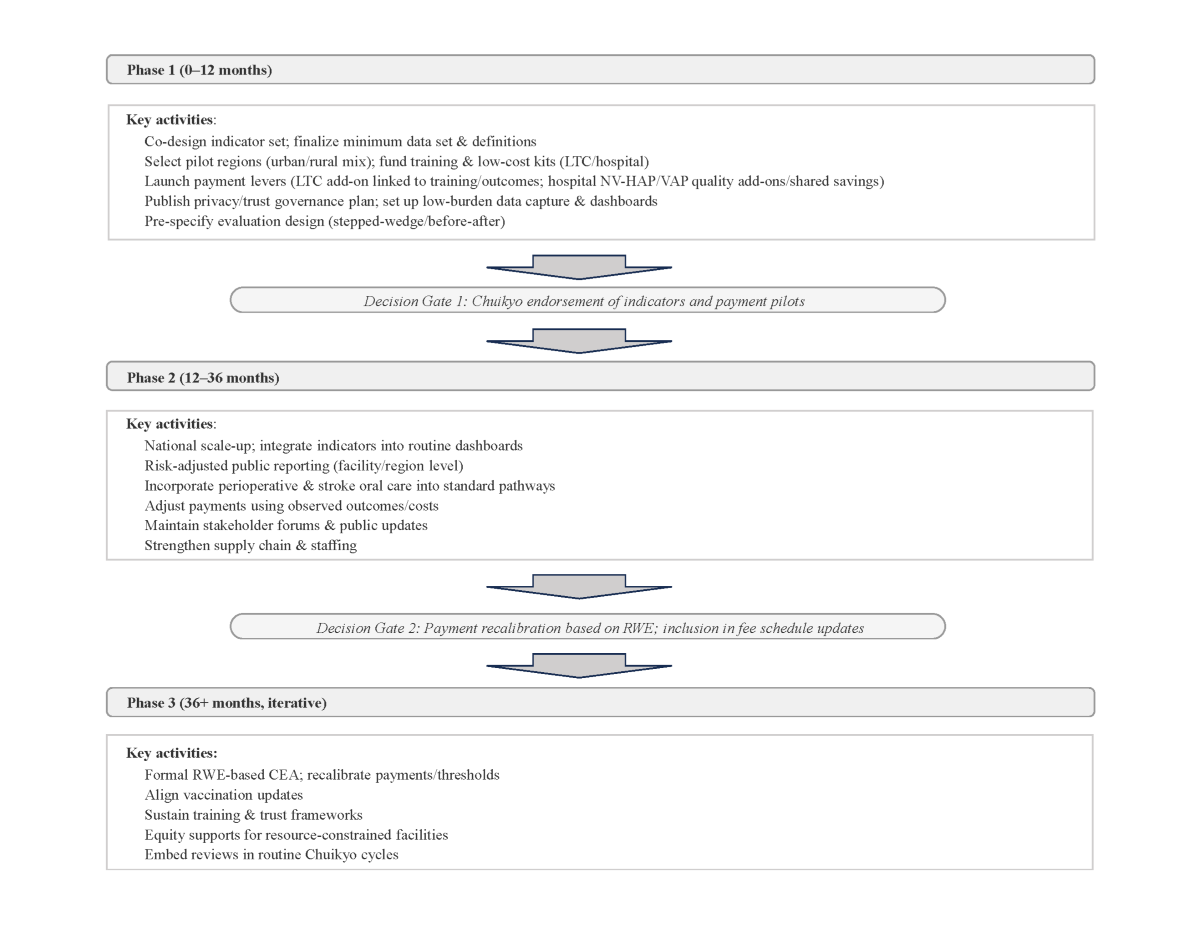
Phased implementation roadmap (12 to 24+ ongoing months). The roadmap outlines a staged implementation plan for an indicator-driven, value-based pneumonia prevention architecture. Phase 1 (0-12 months) focuses on co-designing indicators and minimum datasets, selecting pilot regions, funding training and low-cost oral-care resources, launching initial payment levers, and establishing privacy/trust governance and low-burden data capture. Decision gate 1 marks Chuikyo endorsement of the indicator set and payment pilots. Phase 2 (12-36 months) scales up routine dashboards and risk-adjusted reporting, standardizes perioperative and stroke oral-care pathways, and refines payment parameters using observed outcomes and costs. Decision gate 2 reflects payment recalibration based on real-world evidence and potential inclusion in fee schedule updates. Phase 3 (36+ months) institutionalizes iterative evaluation, real-world-evidence–based cost-effectiveness analysis, periodic recalibration, and equity supports. CEA: cost-effectiveness analysis; Chuikyo: Central Social Insurance Medical Council; LTC: long-term care; NV-HAP: nonventilator hospital-acquired pneumonia; RWE: real-world evidence; VAP: ventilator-associated pneumonia.

A feasible path begins with a 12‑month pilot that co‑designs indicator specifications with public posting and version control; defines a minimum dataset and data flows leveraging claims–EHR linkage; automates antibiotic DOT extraction where possible; selects pilot regions that include an urban–rural mix; funds staff training and low‑cost oral‑care kits; and launches adherence‑focused add‑ons under a prespecified evaluation design such as a stepped‑wedge or controlled before–after design [15,17,22]. To address confounding and site heterogeneity, pilot sites will be stratified by key characteristics (eg, urban/rural context and facility type), and roll-out timing may be randomized where feasible (stepped-wedge). Analyses will incorporate prespecified risk adjustment and time effects (including seasonality) to support fair within-site and between-site comparisons. Deliverables include a data-protection impact assessment and standardized data-use agreements consistent with international recommendations [18], POA education materials and audits, a validation protocol for NV-HAP definitions, and a brief staff workload/acceptability assessment (eg, short staff surveys and time-burden checks) to ensure feasibility alongside fidelity monitoring. Chuikyo endorsement and publication of methods serve as the first decision gate [9,10]. Over the subsequent 24 months, national scale‑up introduces dashboards and risk‑adjusted public reporting with equity safeguards, standardizes perioperative and stroke oral‑care pathways, and recalibrates thresholds, risk adjustment, and payment levels by using pilot real‑world evidence, with technical assistance for facilities serving complex populations [13,20,21]. Thereafter, real‑world evidence‑based cost‑effectiveness, periodic recalibration, and alignment of vaccination policy with the broader prevention package are institutionalized (Figure 2).

### Governance, Privacy, Trust, and Equity

Data linkage and payment experimentation require trust. Governance should treat transparency, patient and public engagement, and privacy‑by‑design as first‑class requirements. Reporting burden is contained by automating data extraction from EHRs and administrative systems; using POA fields and device‑day denominators to attribute outcomes fairly; and explaining public reports in plain language [14,15,18,22]. Equity safeguards include outreach to rural and disadvantaged areas, flexible delivery in long‑term care, and supplemental support for providers caring for complex patients. Public communication should emphasize that comparisons are risk‑adjusted and that thresholds are periodically recalibrated under published rules rather than ad hoc pressure [17].

### Anticipated Impact and Economic Value

When implemented together, vaccination and complementary prevention can produce measurable gains with favorable economics. NV‑HAP and VAP fall when oral care, mobility, and ventilator bundles are implemented with fidelity [4,14-16]; postoperative and stroke‑associated pneumonia decline with perioperative oral health management and standardized oral hygiene plus dysphagia coordination [13,20]; and antibiotic DOT decreases as infections are prevented rather than merely managed, reinforcing stewardship goals [22,26]. Length of stay and readmissions should fall accordingly. Under plausible baseline rates and Japanese unit costs, these changes are likely to be cost-effective and may be cost-saving, particularly when reductions in pneumonia events translate into fewer excess bed-days. The illustrative budget impact scenario (Multimedia Appendix 2) makes assumptions explicit and includes an unfavorable-case scenario, in which results approach break-even, highlighting dependence on baseline incidence, excess length of stay, unit costs, and implementation costs. As programs mature, incremental cost‑effectiveness ratios improve because fixed training costs are amortized, adherence rises, and feedback loops accelerate learning.

### Limitations

This viewpoint synthesizes published evidence and policy documents rather than new empirical analyses. Effect sizes for vaccines and complementary interventions vary by population, setting, baseline risk, and implementation fidelity, and some bundle components such as oral chlorhexidine in ICU settings remain context-dependent and may have heterogeneous effects across institutions [14,16,19]. As a result, the magnitude of benefits described here should be interpreted as plausible ranges rather than fixed expectations for every site.

Second, our indicator proposals rely primarily on routinely collected administrative and electronic health record data. While this approach lowers burden and enables scale, claims‑based risk adjustment cannot fully substitute for clinical granularity. Misclassification of NV‑HAP is a recognized challenge; algorithms based on diagnosis codes, antibiotic starts, and imaging orders may be sensitive to ordering practices and documentation culture. The availability and consistency of present‑on‑admission fields, stroke severity measures (eg, National Institutes of Health Stroke Scale/Score), dysphagia screens, and medication administration records will influence precision [20,22,23]. We propose chart‑review validation in a sample at each pilot site and public version control of indicator logic to mitigate these risks, but residual bias is possible.

Third, data linkage and public reporting entail governance and privacy risks. Although we outline a data‑protection impact assessment, standardized data use agreements, and transparency measures aligned with international recommendations [18], stakeholder trust depends on consistent practice, clear communication, and visible redress mechanisms. Differences in local EHR architectures and data quality may also create inequities in reporting capability; technical assistance will be required to avoid penalizing resource‑constrained facilities.

Fourth, payment levers may generate unintended consequences if poorly calibrated. Paying for adherence may shift attention toward measured processes at the expense of unmeasured but important elements of care (“gaming” or checklist fatigue). Shared‑savings models can create selection incentives if risk adjustment is inadequate, and savings attribution is complicated by concurrent initiatives. To limit these risks, the design emphasizes verification, conservative sharing percentages, risk‑adjusted peer comparisons, and periodic recalibration using real‑world evidence, but some behavioral responses are difficult to anticipate. These sampled chart-review validations also function as an active safeguard against misclassification and gaming by flagging discrepancies between algorithm-reported indicators (and documented bundle adherence) and clinical documentation, triggering feedback, specification updates, and—where applicable—payment recalibration.

Fifth, the budget impact scenario is illustrative. We selected conservative but still uncertain assumptions for baseline NV‑HAP/VAP rates, excess length of stay, and unit costs. Local unit prices, staffing costs, and baseline performance will shift the crossover point between budget neutrality and savings. Additional benefits from perioperative oral health management and dysphagia screening were intentionally excluded from the base case and would likely improve the fiscal picture, but we do not claim precise net effects absent in site‑specific modeling [13,20,24].

Sixth, generalizability has boundaries. Several enablers are specific to Japan—Chuikyo fee schedule cycles, the C2H methodological framework, the Diagnosis Procedure Combination database, and the evolving use of the My Number insurance card for health data linkages [9-11,21]. We argue that the core logic is portable (computable indicators with POA rules, staged incentives, low‑burden data flows, and adaptive recalibration); yet, transfer to other systems will require tailoring to local legal, technical, and workforce contexts.

Finally, implementation capacity is a real constraint. Reliable oral care and mobility require protected time, training, and supplies; ICU bundles depend on interprofessional coordination; and long‑term care facilities vary widely in staffing and access to dental professionals. Up‑front investment and change management may be difficult during workforce shortages. The phased plan explicitly funds training and low‑cost kits and provides technical assistance, but timelines may need adjustment in settings with severe resource limitations.

These limitations motivate the formative design itself: public, version‑controlled indicator specifications; validation against clinical review; equity safeguards in reporting; conservative payment parameters; and iterative recalibration based on real‑world evidence.

## Conclusions

A vaccination‑centered prevention strategy can deliver its promised value only when surrounded by reliable measurement, transparent governance, practical payment incentives, and bedside prevention that addresses aspiration risk and hospital‑acquired pneumonia. Japan has the ingredients to make this link credible and visible: an established HTA framework; improving interoperability through the My Number insurance card; and professional capacity in nursing, dentistry, infection prevention, geriatrics, and informatics. A formative, indicator‑driven architecture that ties vaccination to oral‑care–based prevention and value‑based payment can reduce avoidable harm and spending, preserve function and dignity, and strengthen public confidence. The approach is testable now and generalizes to other vaccine‑preventable syndromes where bedside practice, program design, and financing interact.

## Appendix

Multimedia Appendix 1 Summary of the clinical effectiveness, economic value, implementation keys, and candidate indicators by intervention and setting.

Multimedia Appendix 2 Simple budget impact scenario.

## Abbreviations

C2H: Center for Outcomes Research and Economic Evaluation for Health
Chuikyo: Central Social Insurance Medical Council
DOT: days of therapy
EHR: electronic health record
HTA: health technology assessment
ICU: intensive care unit
NV‑HAP: nonventilator hospital‑acquired pneumonia
POA: present-on-admission
VAP: ventilator‑associated pneumonia

## Data Availability

No new datasets were generated or analyzed. All sources cited are publicly available.
