# Supplementary material for: Designing an Indicator‑Driven, Value‑Based Architecture for Pneumonia Prevention in Japan: A Formative Policy Viewpoint on Adult Vaccination and Oral Care
Source: JMIR Form Res. 2026 Feb 27;10:e86912. doi: 10.2196/86912 (PMC12988348; doi:10.2196/86912)
Supplement: Multimedia Appendix 1 [file formative_v10i1e86912_app1.docx]

Appendix 1. Indicator specifications

**Table S1. Summary of clinical effectiveness and economic value by intervention and setting**

| **Intervention** | **Care setting/target** | **Main clinical effect** | **Economic value** | **Japan-specific evidence** | **Implementation keys** | **Candidate KPIs** | **Key references** |
| --- | --- | --- | --- | --- | --- | --- | --- |
| Professional + mechanical oral care in nursing homes | LTC, high-risk older residents | Pneumonia incidence ↓; mortality risk ↓ in high-risk groups | Cost-effective (Japan) | Yes (13) | Dentist/hygienist visits; caregiver daily care; oral care kits; training and supervision | Aspiration pneumonia per 100 residents-years; acute transfers; antibiotic days; oral status assessment completion | 3,4,13,29 |
| Perioperative oral management/hygiene | Pre- and postoperative (e.g., thoracic, esophagectomy, cancer surgery) | Postoperative pneumonia ↓; respiratory infections ↓ | Cost-effective (Japan; downstream costs) | Yes (14) | Protocolized dental/hygiene consult; ERAS integration; supplies; documentation | 30-day postoperative pneumonia; LOS; ICU admissions; protocol adherence | 2,14,15 |
| NV-HAP prevention (nurse-driven oral care/mobility/aspiration precautions) | Adult inpatient wards (non-ventilated) | NV-HAP ↓; antibiotic use and LOS often ↓ | Often cost-saving (ROI reported) | Indirect; international programs (10,11,20); narrative review (9) | Standardized kits; missed-care monitoring; staffing/time protection; education | NV-HAP per 1,000 patient-days; oral care adherence; missed oral care opportunities; antibiotic days | 9,10,11,20 |
| VAP bundle with oral decontamination (chlorhexidine where indicated) | Adult ICU, mechanically ventilated | VAP ↓; ventilator days sometimes ↓ | Often cost-saving | No direct Japan CEA; international studies (16–19) | Bundle checklist; CHG availability; suction devices; nurse/RT roles | VAP per 1,000 ventilator-days; ventilator days; bundle adherence; antimicrobial days | 16–19,30 |
| Stroke-unit standardized oral hygiene | Acute stroke wards | Potential SAP ↓; improved comfort/secretion management | Uncertain | Not yet; international pilot/feasibility and NICE review (21–23) | Integrate into stroke pathways; nurse training; dysphagia coordination | Stroke-associated pneumonia within 7 days; oral care completion; dysphagia screen within 24h | 21–23 |
| Targeted vaccination for older adults | Community-dwelling older adults at risk | CAP hospitalizations and severe outcomes ↓ | Cost-effective (international); governed by CEA/budget impact in Japan | Burden and policy process documented (1,5) | Coverage tracking; outreach; co-pay supports as needed | Vaccine coverage rates; CAP hospitalizations per 1,000 older adults; outpatient CAP episodes/costs | 1,5 |

Note: Economic value categories are defined as follows: dominant, better outcomes at lower costs; cost-effective, improved outcomes at an acceptable incremental cost (ICER, eg, per QALY) versus alternatives; cost-saving, net cost reduction with noninferior or improved outcomes; uncertain, insufficient or mixed evidence. Several NV-HAP and VAP programs have reported positive return on investment; accordingly, these rows are labeled “often cost-saving (ROI reported).” Abbreviations: CAP: community-acquired pneumonia; CEA: cost-effectiveness analysis; CHG: chlorhexidine gluconate; ERAS: enhanced recovery after surgery; ICER: incremental cost-effectiveness ratio; ICU: intensive care unit; NICE: National Institute for Health and Care Excellence; NV-HAP: nonventilator hospital-acquired pneumonia; ROI: return on investment; R: respiratory therapist; SAP: stroke-associated pneumonia; VAP: ventilator-associated pneumonia.

This appendix provides a minimum, harmonized specification of “what to count” for the indicators described in the main text. A full specification with version control will be published at pilot launch.

1) Pneumococcal vaccination coverage among adults aged 65+

- Numerator: Number of persons aged 65 years or older who received PPSV23 or a pneumococcal conjugate vaccine during the measurement year (de‑duplicate multiple doses; count the most recent dose only).

- Denominator: Number of insured persons aged 65 years or older in the jurisdiction.

- Exclusions: Documented medical contraindications (e.g., prior anaphylaxis).

- Data sources: Municipal immunization registry and claims (vaccine product codes and administration codes).

- Note: Reconcile registry and claims as dual sources to minimize inter‑municipal variation.

2) Timeliness of seasonal influenza vaccination

- Numerator: Number of persons aged 65+ vaccinated for influenza by a pre‑specified pre‑season cutoff date (e.g., December 15).

- Denominator: Number of persons aged 65+ who received an influenza vaccine during the measurement year.

- Data sources: Immunization registry and claims.

3) Incidence of nonventilator hospital‑acquired pneumonia (NV‑HAP)

- Numerator: Number of pneumonia events occurring ≥48 hours after admission and not present on admission (POA). Algorithm requires all of: (a) pneumonia ICD code; (b) new initiation of guideline‑concordant or broad‑spectrum antibiotics; and (c) chest imaging performed. Exclude events while on invasive mechanical ventilation.

- Denominator: Patient‑days on general wards (exclude ICUs).

- Exclusions: Patients receiving end‑of‑life palliative care; extreme immunosuppression may be reported separately in sensitivity analyses.

- Risk adjustment: Age, sex, Charlson comorbidity, emergency vs elective admission, major diagnostic category (MDC), early postoperative status, ward type, proxy for neurologic status (e.g., Japan Coma Scale), and proxies for nutrition/dysphagia risk (e.g., nil per os).

- Data sources: DPC/claims plus EHR (medication start times, imaging timestamps).

- Validation: Structured chart review (target ~50 cases per facility).

4) Incidence of ventilator‑associated events/pneumonia (VAE/VAP)

- Numerator: Events meeting NHSN VAE criteria (VAC/IVAC/PVAP) or clinically adjudicated VAP.

- Denominator: Ventilator‑days.

- Data sources: ICU documentation, EHR, surveillance systems.

- Note: Oral chlorhexidine is not universally recommended; if used based on local indication, an optional process indicator may capture its documented use.

5) 30‑day postoperative pneumonia (selected procedures)

- Numerator: Pneumonia events within 30 days of index surgery for specified procedures (e.g., major gastrointestinal, head and neck, thoracic).

- Denominator: Number of patients undergoing the specified procedures.

- Exclusions: Preoperative pneumonia; emergency procedures.

- Data sources: DPC/claims plus EHR.

- Risk adjustment: Age, comorbidities, surgical risk, intraoperative blood loss, transfusion, operative time, and ICU admission.

6) Stroke‑associated pneumonia within 7 days

- Numerator: Pneumonia events occurring within 7 days of admission or stroke onset among stroke hospitalizations (ischemic or hemorrhagic).

- Denominator: Number of stroke hospitalizations.

- Risk adjustment: Age, sex, stroke severity (NIHSS where available; otherwise JCS or mechanical ventilation as proxies), mechanical ventilation, and documented dysphagia screening.

- Data sources: DPC/claims plus EHR.

7) Antibiotic days of therapy (DOT) per 1,000 patient‑days

- Numerator: Sum of antibiotic days of therapy (one DOT per antibiotic per patient per day, regardless of number of doses).

- Denominator: Patient‑days (ward‑level or ICU‑specific as appropriate).

- Data sources: Medication administration records (preferred). Where unavailable, construct transparent proxies from parenteral administration days and oral prescription days.

- Note: Output variable names should align with the NHSN AUR specification. Clearly flag when proxies are used.

8) Present‑on‑admission (POA) implementation

- Definition: Conditions present at the time the order for inpatient admission occurs and not attributable to inpatient care.

- Implementation: Standardize the DPC POA attribute; provide education materials and audit procedures to ensure consistent POA assignment.
