# Supplementary material for: Designing an Indicator‑Driven, Value‑Based Architecture for Pneumonia Prevention in Japan: A Formative Policy Viewpoint on Adult Vaccination and Oral Care
Source: JMIR Form Res. 2026 Feb 27;10:e86912. doi: 10.2196/86912 (PMC12988348; doi:10.2196/86912)
Supplement: Multimedia Appendix 2 [file formative_v10i1e86912_app2.docx]

Appendix 2. Simple Budget Impact Scenario

**Purpose**

To provide an order-of-magnitude estimate of the fiscal impact of an adherence add-on plus conservative shared savings for nonventilator hospital-acquired pneumonia (NV-HAP) and ventilator-associated pneumonia (VAP) prevention and related practices (oral care, early mobility) in a representative hospital, following ISPOR Budget Impact Analysis principles (target population, perspective, time horizon, cost components, and sensitivity analysis).

**Perspective and time horizon**

Mixed payer–provider perspective over 1 fiscal year.

**Setting**

A 300-bed general hospital with 85% average occupancy, yielding approximately 93,000 patient-days per year; an intensive care unit (ICU) with 10 beds and about 2,000 ventilator-days per year.

**Currency conversion**

All Japanese yen (¥) amounts were converted to US dollars using OANDA (US $1 = ¥153.1; accessed February 14, 2026).

**Baseline rates, effects, and unit costs**

- **NV-HAP baseline incidence:** 1.2 per 1,000 patient-days → about 112 events/year. Relative reduction with bundle adherence: 25% (conservative within published ranges). Excess length of stay (LOS) per event: 4 days.
- **VAP baseline incidence:** 5 per 1,000 ventilator-days → about 10 events/year. Relative reduction: 30%. Excess LOS per event: 6 ICU-equivalent days.
- **Unit costs:** ¥60,000 (US $392) per general ward day; ¥80,000 (US $523) per ICU-equivalent day (illustrative; localize to payer schedule).

**Gross savings**

- **NV-HAP:** 28 averted events × 4 days × ¥60,000 (US $392) = ¥6.72 million (US $43,893).
- **VAP:** 3 averted events × 6 days × ¥80,000 (US $523) = ¥1.44 million (US $9,405).
- **Total gross savings:** ¥8.16 million (US $53,310).

**Incremental program costs (Year 1)**

- **Training, education, and auditing:** ¥0.8 million/year (US $5,225).
- **Minor electronic health record (EHR) configuration:** ¥0.5 million (US $3,266) (one-time).
- **Oral care supplies:** target exposure 30% of patient-days; ¥100 (US $0.65) per patient-day × 27,900 exposed patient-days = ¥2.79 million (US $18,223).
- **Early mobility materials, posters, and staff time for audits:** ¥0.3 million (US $1,960).
- **Total incremental costs:** ¥4.39 million (US $28,673) in Year 1.

**Net impact and incentive sharing**

- **Net savings (payer + provider):** approximately ¥3.77 million (US $24,625) in Year 1 after program costs.
- If 30% of verified, risk-adjusted savings are shared with the hospital, the facility receives about ¥1.13 million (US $7,380), leaving about ¥2.64 million (US $17,245) in payer-side net savings. In Year 2 and beyond, net savings increase as EHR configuration costs fall away and adherence improves.

**Sensitivity analysis (unfavorable case)**

- **NV-HAP baseline** 0.8/1,000 patient-days; **relative reduction** 20%; **unit cost** ¥50,000 (US $327)/day → gross savings about ¥3.20 million (US $20,903). With the same program costs, the overall budget impact approaches break-even to a small deficit. Neutrality can typically be restored by lowering supply unit costs, narrowing the initial target population, or internalizing training.

**Notes**

- The scenario excludes additional gains from perioperative oral management and standardized dysphagia screening in stroke units; these would add to savings.
- In long-term care, professional/mechanical oral care for high-risk residents is generally cost-effective; near-term budget impact may be neutral to slightly net cost depending on wages and kit prices. Designing the add-on alongside readmission avoidance and targeted vaccination can improve near-term fiscal performance.
- Counts are rounded to the nearest whole number for presentation.
